# An IoT-based smart mosquito trap system embedded with real-time mosquito image processing by neural networks for mosquito surveillance

**DOI:** 10.3389/fbioe.2023.1100968

**Published:** 2023-01-20

**Authors:** Wei-Liang Liu, Yuhling Wang, Yu-Xuan Chen, Bo-Yu Chen, Arvin Yi-Chu Lin, Sheng-Tong Dai, Chun-Hong Chen, Lun-De Liao

**Affiliations:** ^1^ National Mosquito-Borne Diseases Control Research Center, National Health Research Institutes, Zhunan Township, Taiwan; ^2^ Institute of Biomedical Engineering and Nanomedicine, National Health Research Institutes, Zhunan Township, Taiwan; ^3^ Department of Biotechnology and Bioindustry Sciences, National Cheng Kung University, Tainan, Taiwan; ^4^ National Institute of Infectious Diseases and Vaccinology, National Health Research Institutes, Zhunan Township, Taiwan; ^5^ Institute of Molecular Medicine, College of Medicine, National Taiwan University, Taipei, Taiwan

**Keywords:** mosquito-borne diseases, *Aedes aegypti*, *Culex quinquefasciatus*, computer vision technology, deep learning

## Abstract

An essential aspect of controlling and preventing mosquito-borne diseases is to reduce mosquitoes that carry viruses. We designed a smart mosquito trap system to reduce the density of mosquito vectors and the spread of mosquito-borne diseases. This smart trap uses computer vision technology and deep learning networks to identify features of live *Aedes aegypti* and *Culex quinquefasciatus* in real-time. A unique mechanical design based on the rotation concept is also proposed and implemented to capture specific living mosquitoes into the corresponding chambers successfully. Moreover, this system is equipped with sensors to detect environmental data, such as CO_2_ concentration, temperature, and humidity. We successfully demonstrated the implementation of such a tool and paired it with a reliable capture mechanism for live mosquitos without destroying important morphological features. The neural network achieved 91.57% accuracy with test set images. When the trap prototype was applied in a tent, the accuracy rate in distinguishing live *Ae. aegypti* was 92%, with a capture rate reaching 44%. When the prototype was placed into a BG trap to produce a smart mosquito trap, it achieved a 97% recognition rate and a 67% catch rate when placed in the tent. In a simulated living room, the recognition and capture rates were 90% and 49%, respectively. This smart trap correctly differentiated between *Cx. quinquefasciatus* and *Ae. aegypti* mosquitoes, and may also help control mosquito-borne diseases and predict their possible outbreak.

## Introduction

Mosquitoes have been responsible for the spread of several kinds of viruses among human populations for a long time. Recent mosquito-borne virus outbreaks, including dengue fever, chikungunya, and Zika virus, have affected many people (Gubler 2002; Roth et al., 2014; Patterson et al., 2016; Mayer et al., 2017). The spread of these diseases has become more prevalent due to recent expansions in the geographical distributions **of** mosquito species that serve as vectors. *Aedes* (*Ae.*) *aegypti* and *Ae. albopictus*, two species of mosquitoes responsible for spreading viruses, such as dengue and chikungunya viruses, are now widely distributed in tropical and subtropical areas, putting approximately half of the world’s population at risk of infection (Bhatt et al., 2013; Kraemer et al., 2015).

*Ae. aegypti* and *Ae. albopictus* are the two species of mosquitoes that are primarily responsible for the spread of dengue fever in Taiwan (Lei et al., 2002). The distributions of these species differ in Taiwan. The former species is distributed in the south of Taiwan (south of the Tropic of Cancer, 23º35′N), whereas the latter is found throughout the island (Chen, 2018). *Ae. aegypti* originated in Africa but is now prevalent in most tropical and subtropical regions of the world (Tabachnick, 1991). *Ae. albopictus* is endemic to Southeast Asia. The distribution of this species has now expanded, with broad coverage across the world, including Europe and Africa (Hawley et al., 1987; Bonizzoni et al., 2013). Therefore, both species of *Aedes* mosquitoes are highly likely to be alien species in Taiwan. Studies have shown that dengue has apparently been epidemic in Taiwan since 1872 (Koizumi, 1917). Thus, it can be inferred that *Aedes* mosquitoes capable of transmitting dengue fever existed in Taiwan in 1872. Sporadic dengue outbreaks have also occurred, but outbreaks of dengue fever have continuously been recorded since the 1980s (Lin et al., 2010; Wang et al., 2016). To control and mitigate dengue fever outbreaks, one must have sufficient information about the spread and distribution of *Ae. aegypti* and *Ae. albopictus*. In other countries affected by mosquito-related diseases, mosquito surveillance systems are often implemented to predict and mitigate mosquito-borne diseases. The data obtained by mosquito surveillance systems have been shown to correlate with the number of human infections, and such a system may serve as a valuable tool for evaluating the risk of mosquito-borne virus infections (Kilpatrick and Pape, 2013). Thus, it is vital to implement a system targeting *Ae. aegypti* and *Ae. albopictus* to control possible future outbreaks of dengue fever.

The traditional method of mosquito surveillance is to install several mosquito traps throughout the surveilled area to capture live mosquitoes, which can then be transported to a laboratory for identification and analysis (Andreadis et al., 2001). However, currently available mosquito traps are not effective under real-world conditions for the following reasons. First, conventionally used mosquito traps collect all types of insects without discriminating whether the insect can transmit disease. The laborious work of separating mosquitoes from the mosquito traps must be done manually in the laboratory. Second, conventional traps unable to collect environmental data, such as temperature or humanity, when such variables have been shown to be correlated with the potential distribution of mosquitoes (Neteler et al., 2011). Clearly, mitigating the spread of mosquito-borne diseases requires a multifunctional mosquito trap design.

New kinds of mosquito traps have been proposed; for example, Microsoft has initiated a research project called Project Premonition to develop a new robotic trap (Linn 2016a; Linn 2016b). Their new mosquito trap design aims to collect only the mosquitoes targeted for tracking and recording critical environmental data, such as trap temperature, wind speed, and humidity. New technologies have been employed to improve the performance of this mosquito trap. An image recognition system equipped with a pre-trained neural network enables the mosquito trap to recognize whether the insect in the trap is the mosquito species of interest. With this cutting-edge technology, it can be anticipated that the recognition ability of the mosquito trap will produce great results, relieving researchers from burdensome mosquito identification and sorting. While the future of this project seems promising, the cost of the mosquito trap may not be inexpensive in a wide range of regions, given its complex design and hardware requirements.

*Ae. aegypti* and *Ae. albopictus* have similar phenotypic characteristics, and there are black and white stripes on the bodies of *Aedes* mosquitoes. Unless it is necessary to clearly distinguish between *Ae. aegypti* and *Ae. albopictus*, the black and white stripes on the body are sufficient for use as the basis for identifying *Aedes* mosquitoes. In contrast, *Culex* mosquitoes do not have obvious black and white stripes, so it is a great trait for easily discerning between these two different mosquito species. Here, a new mosquito trap concept is proposed to detect *Aedes* and non-*Aedes* mosquitoes *via* image processing using multiple deep learning networks and a fully automatic trap system. The aim of this mosquito trap research was to effectively collect living target mosquitoes and obtain valuable environmental data *via* the trap, while maintaining a low cost of manufacturing and implementation. The design allows the real-time detection of mosquitoes in a single setting. It uses a specifically trained, fully connected neural network to target the two major species of mosquitoes responsible for the spread of dengue fever in Taiwan, *Ae. aegypti* and *Ae. albopictus*. This mosquito trap system can also record the CO_2_ concentration, humidity, and temperature of the surrounding environment when a mosquito is captured. These data, along with the time and location where the mosquito is captured, are uploaded to a cloud database for real-time observation. With this mosquito trap design, valuable functionality is provided at a lower cost, allowing the broad implementation of a mosquito surveillance system, that is, both effective and affordable.

## Material and methods

### Design of the smart mosquito trap system

**FIGURE 1:**
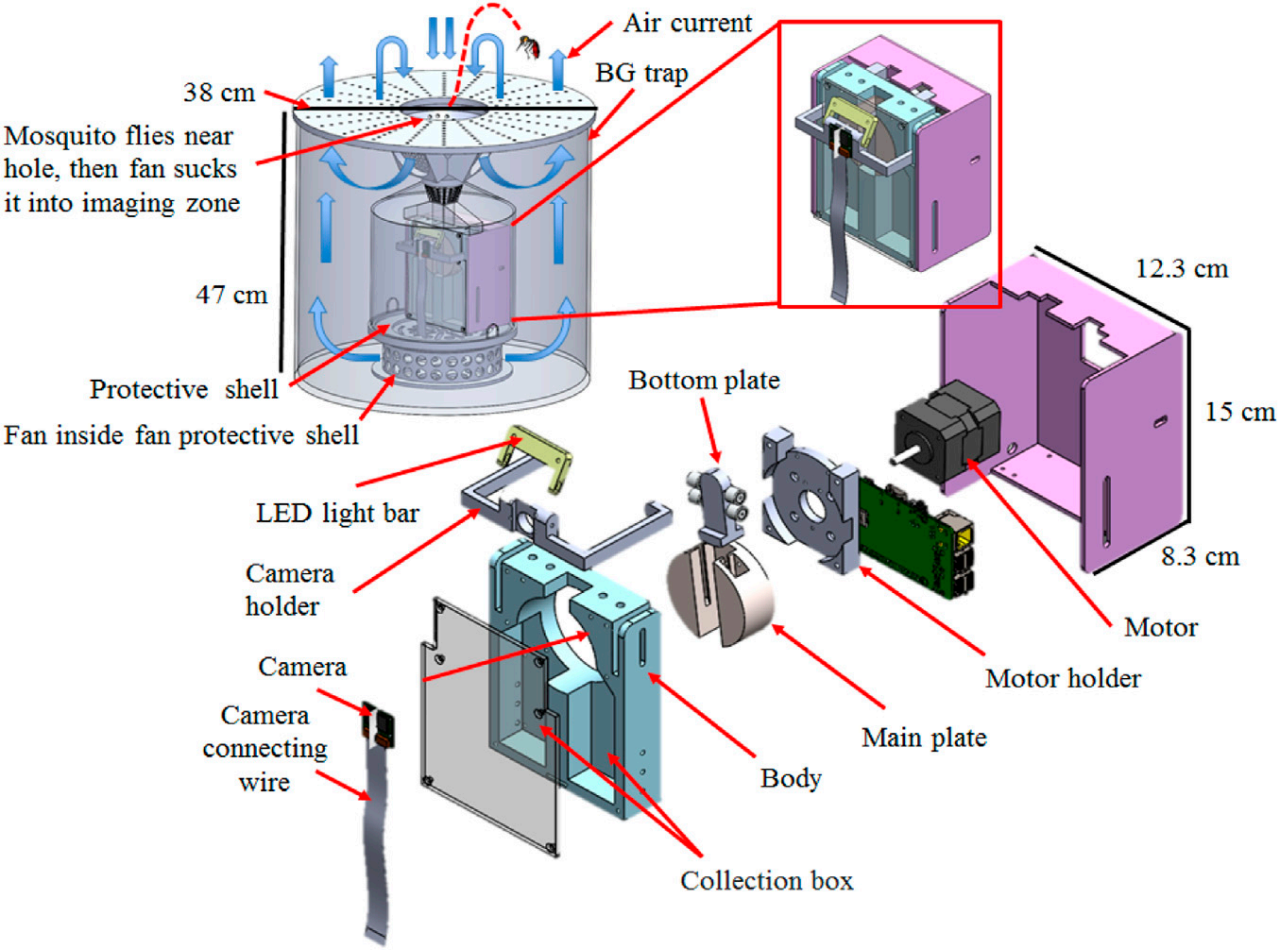
Schematic diagram of the developed mosquito trap. The large fan at the bottom of the BG trap pushes the air upward, and the three fans at the upper entrance pull the air. The air exits through the outer edge of the 38-cm-diameter lid and enters through the center. Therefore, when mosquitoes fly near the BG trap, they are sucked in by the trap’s airflow. The core device mechanism and operating process of the mosquito trap are described in the Materials and Methods section. The mosquito trap includes two compartments (collection boxes): one on the left for collecting *Aedes* mosquitoes; and one on the right for collecting *Culex* (non-*Aedes*) mosquitoes.”

The core device of the mosquito trap has two chambers (collection boxes), one of which collects mosquitoes determined to be *Ae. aegypti* or *Ae. albopictus* and the other collects mosquitoes that are determined to be *Culex* (*Cx.*) *quinquefasciatus* (Figure 1). Above these two chambers, a circular capture plate is paired with a camera that captures images of mosquitoes entering the bottom two chambers. The capture plate is made of the following components: 1) the main plate, 2) a sliding guide and an attached bottom plate, and 3) a stepper motor.

**FIGURE 2:**
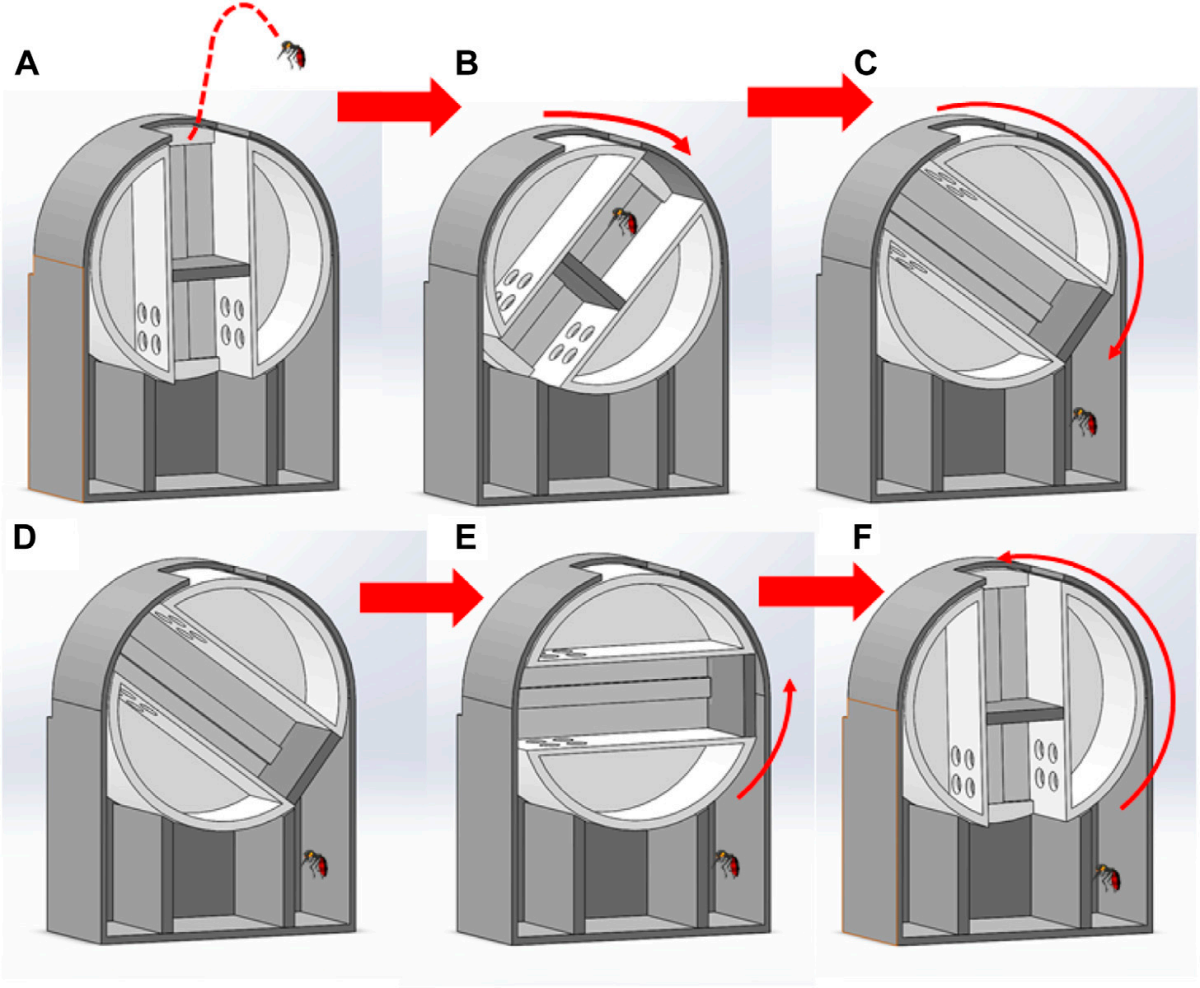
The mosquito trap uses a rotation method to capture and classify mosquitoes. **(A)** Mosquitoes fly into the capture area. **(B)** The mechanism rotates, blocking the opening. **(C)** After the mechanism rotates to a certain angle, the rotation stops, and the chassis pushes the mosquitoes into the corresponding chamber. **(D)** The plate remains in the same position for a period of time. **(E)** The plate will then rotate in the opposite direction. **(F)** The plate rotates and returns to its original position.

The main plate is in the form of a cylinder on which a linear track is etched to place the sliding guide. The main plate provides an area (the capture area) for the mosquito to enter the trap and undergo detection (mosquito detection is described in detail in the image recognition system section). The main plate also rotates when needed when a mosquito enters the trap (Figure 1 and Figure 2). The sliding guide is placed on the main plate’s linear track, with a bottom plate attached to the part that slides freely on the linear track. When the sliding guide slides to its limit on one side, an area forms between the track and the top of the bottom plate; this area is called the capture area. Mosquitoes can fly into this area and undergo detection. When the sliding guide slides the bottom plate to its limit on the other side, it closes off the capture area and pushes the mosquito, if present, from the capture area to the top of the track.

A stepper motor is attached to the main plate through the center of the circle. The stepper motor rotates the base plate when the mosquito trap detects target mosquitoes that fly into the capture area. There is a ceiling with an opening in the middle above the capture plate. This ceiling comprises an area identical to that of the capture plate and leaves the mosquito nowhere to go except through the top opening. The ceiling helps prevent mosquitoes from escaping during capture.

The core device of the mosquito trap was placed into a BG trap (Biogents™, Regensburg, Germany). Mosquito attractants (Guangzhou Baikong Biological Co., Ltd., China) are placed on the BG trap cover or inside the BG trap itself to lure mosquitoes into the mosquito trap capture area (Figure 1). The original BG trap fan is replaced with an AC110V fan (17251A1-HBAPL-TC-N, Sunta, Taiwan), and it is protected by a casing to prevent foreign objects from contacting and breaking the fan blades and entanglement of the wire from the sensors. The mosquito trap was also equipped with a carbon dioxide sensor module (MG811, Sandbox Electronics, Finland) to detect CO_2_ concentration and other sensors to detect humidity and temperature (DHT22, Aosong Electronics Co., Ltd., China), as shown in Supplementary Figure S1. A Raspberry Pi microcontroller (Module B, Raspberry Pi Foundation, England) is used to control all of the electrical components. Once a mosquito enters the entry port and is identified with the image recognition software, as described below, the mosquito species is then classified according to the phenotypic characteristics and collected in the respective chamber (collection box) (Supplementary Figure S2).

### Capture mechanism

When the mosquito trap is not actively trapping mosquitoes, the empty rectangular track on the capture plate aligns with the ceiling opening. The sliding guide’s base plate is at its lowest limit, forming a space in the rectangular track between the top of the sliding guide and the ceiling opening. This area is called the capture area. When a mosquito is detected in the capture area, the capture plate is then rotated by the motor controlled by the Raspberry Pi, which immediately closes the opening, trapping the mosquito in the capture area. When the capture plate rotates more than 90°, the sliding guide’s base plate is lowered by gravity. When the bottom plate starts gliding along with the sliding guide, the capture area decreases and forces the mosquito to move in response to the capture plate (Supplementary Figures S2A,B). Finally, the bottom plate reaches its upper limit. There is no space left in the capture area, and thus the mosquito is forced inside the storage chamber (Supplementary Figure S2C).

After capturing the mosquito, the capturing plate rotates in the opposite direction back to its original position, as confirmed by a positioning sensor (TCST2103, Vishay Intertechnology, United States), as shown in Figures 2D, F. The bottom plate is then again lowered by gravity until it reaches its lowest limit. Finally, the capture area is restored, and the trap is ready to catch another incoming mosquito (Figure 2F). Since the capture mechanism requires rotation of the capture plate up to 180°, the capture plate can be controlled to rotate in different directions according to the detection of different types of mosquitoes, thus achieving the goal of effectively sorting different types of mosquitoes into different chambers.

### Image recognition system

The image recognition tool is essential for the proper functioning of the proposed mosquito trap. The image recognition tool determines whether a mosquito is present in the entry box and, if so, whether it is an *Ae. aegypti* or *Cx. quinquefasciatus* mosquitoes. The algorithm is initiated when motion is detected. To detect the motion, the difference between images from successive timeframes is analyzed to determine whether the movement has occurred; then, the contours of the different images are detected, and for each contour form, a subimage is fed into the neural network. This whole process is implemented with OpenCV. Next, a neural network, that is, sufficiently fast and accurate in detecting the types of mosquitoes is developed on the Raspberry Pi device. We developed two imaging platforms (i.e., static and living imaging platforms) to effectively collect mosquito image data to train our neural network and adapted the SqueezeNet model (Iandola et al., 2016) as our neural network model, where we utilize the method of transfer learning to train our recognition system. The deployed algorithm incorporates OpenCV for motion detection and our trained neural network to recognize mosquitoes in real-time using the Raspberry Pi.

### Database development

**FIGURE 3:**
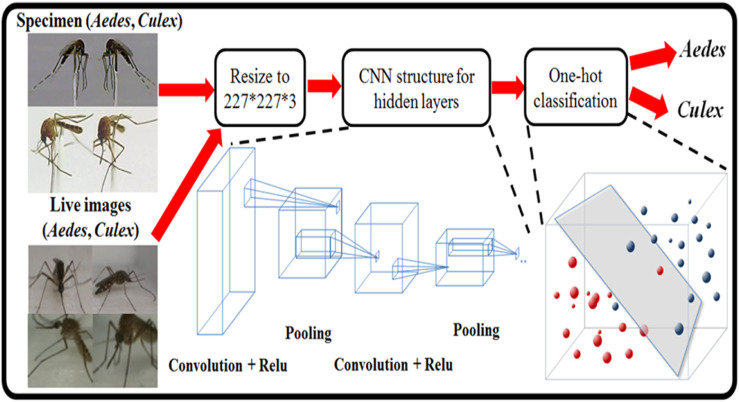
The CNN was trained on captured mosquito images and feature points through convolution. However, with increasing pixel size of the image, number of feature pixels, and number of features found, the computational cost dramatically increases. Therefore, the pooling method was used to compress the images while retaining essential image features to increase performance. Finally, one hot encoding classification was performed to differentiate *Ae. aegypti* and *Cx. quinquefasciatus*.

A neural network trained to differentiate between different mosquitoes is developed to complete the image recognition process (Figure 3). The development of the neural network consists of three significant steps. First, an imaging system is employed to obtain training data by imaging different mosquitoes. Such a system is used to obtain sufficient training data by imaging different static or live mosquito specimens. Finally, the obtained data are fed into an appropriate neural network model for transfer learning.

Due to its success in the ImageNet competition, AlexNet has become one of the most popular research tools since 2012. We use the SqueezeNet neural network to develop the mosquito image recognition system. It is a lightweight and efficient convolutional neural network (CNN) model with 50 times fewer parameters than AlexNet, but the model performance (such as accuracy) is close to that of AlexNet. Small models have many advantages, such as reducing the number of calculations during model training and predictive identification. The speed of a single step is faster, and there are fewer parameters than in a larger model; therefore, the video memory occupied by the network is smaller. The smaller model is beneficial in field-programmable gate array (FPGA) and embedding type device deployment. The proposed mosquito trap uses edge computing to directly perform image recognition of mosquitoes with the system embedded in the mosquito trap. No additional computer or server is needed. Therefore, the model and calculation volume should not be too large or compressed. The model method mainly comprises network pruning, quantization, and knowledge distillation. To reduce the amount of calculation (3 × 3 × number of input channels x number of filters), in the CNN, 3 × 3 convolution kernels (filters) are mostly replaced with 1 × 1 convolution kernels (filters). This concept effectively reduces the parameters by nine times while retaining the accuracy of model identification and then reduces the input of 3 × 3 convolution kernels. The number of input channels dramatically reduces the number of convolution operations. The first layer is a standard convolution base layer (Conv) in terms of structure, with nine fire modules in the middle. The fire module divides the original convolutional layer into two layers, including the squeeze layer of 1 × 1 convolution filters and 1 × 1. It comprises an expanding layer of 3 × 3 convolution filters interspersed with max pooling layer compression parameters, retains features, and prevents interference. After connecting a convolutional layer to control the size of the input and output, global average pooling is used instead of fully connected layers to reduce parameters and reduce overfitting. According to the literature, SqueezeNet can reduce the parameters by 50 times while retaining the same accuracy as AlexNet.

### Imaging of specimens

The first imaging system we developed allowed us to take pictures of mosquitoes in two directions at different angles (Supplementary Figure S3) and use a MATLAB-based graphical user interface (GUI) (Supplementary Figure S3A and Supplementary Movie S1) to set parameters and perform imaging operations. The system consisted of a platform that could be rotated along the *z*-axis using a stepper motor (17hs1910-p4170, JIALIBAO Electronics, China) and rotated 360° (Figure 3B). Mosquitoes were attached to a platform by insect pins. A camera bracket fixed the camera and rotated it along the *x*-axis (Figure 3B). However, due to platform limitations, the rotation angle was limited to 160° (Figure 3B).

On both sides of the camera, a controllable light-emitting diode (LED) (61-238/LK2C-B50638F6GB2/ET, Everlight Electronics Co. Ltd., Taiwan) was designed and placed to achieve necessary lighting conditions. The entire system was placed in a studio with a white background to ensure that the captured images contained only the target mosquito. Some image samples obtained from this imaging system are shown in Figures 3C, D. Different types of mosquito samples were placed on the platform for photographing, and these images formed the basis of the dataset used to train the network (Supplementary Figure S4).

### Imaging of *in vivo* mosquitoes

**FIGURE 4:**
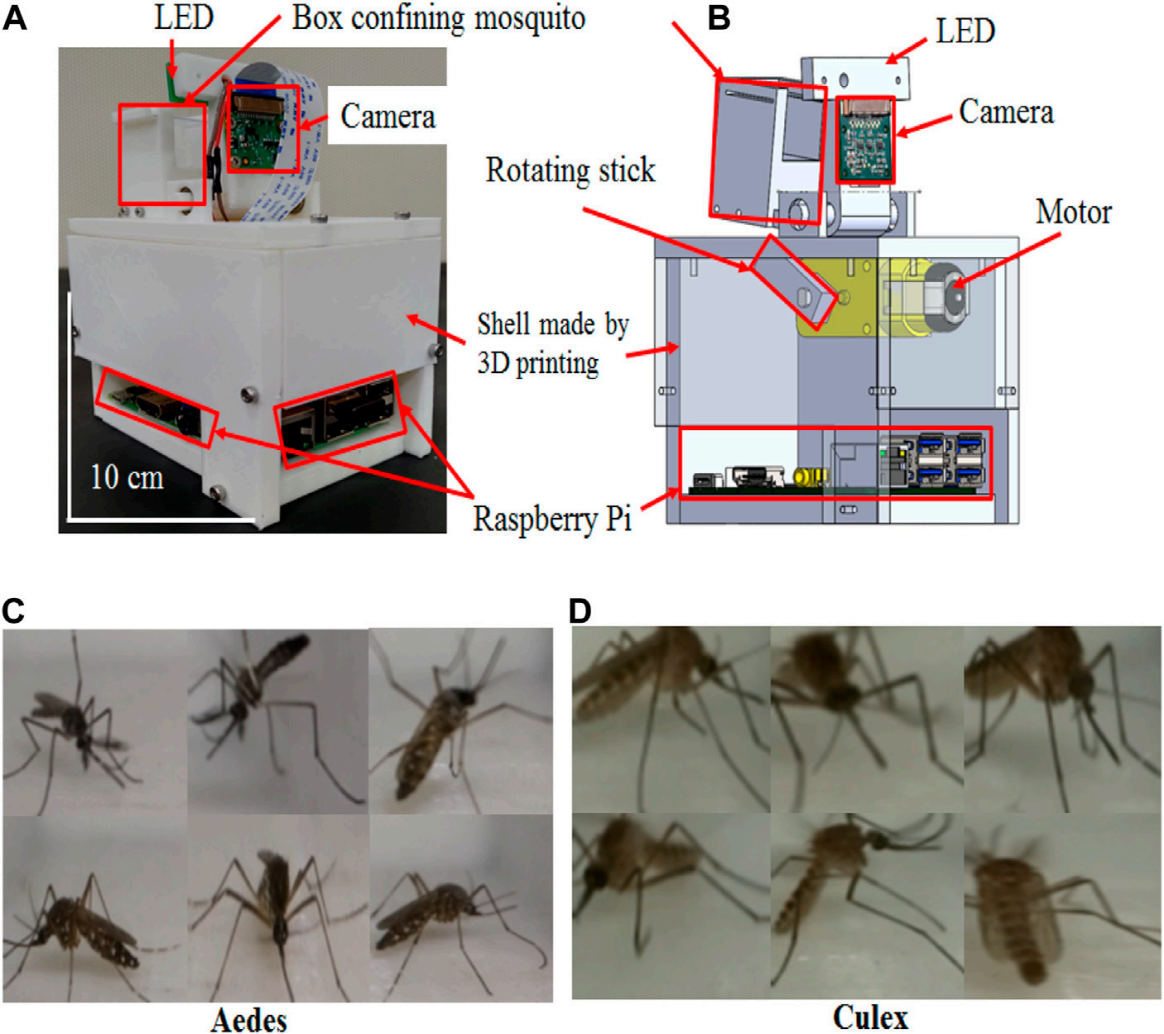
Dynamic images were used in the validation phase during the development of the model used in this study. **(A)** Mechanism of the dynamic mosquito studio. **(B)** Inside perspective of the dynamic mosquito camera. **(C)** Actual images of live females of *Ae. aegypti* and **(D)** *Cx. quinquefasciatus*; females were photographed using a digital camera inside the dynamic mosquito studio based on the Arduino controller.

In addition to obtaining fixed specimen images for neural network training, we also developed a system to obtain live mosquito images to improve the database used for neural network training. The setup resembled the capture area of our mosquito trap (Figure 4A) and included a small, 3D-printed (3030K, Kingssel, Taiwan and Delta, FLUX, Taiwan) box, a camera (Raspberry Pi camera V2, Raspberry Pi Foundation, United Kingdom) and an LED (61-238/LK2C-B50638F6GB2/ET, Everlight Electronics Co., Ltd., Taiwan). The small box, camera, and LED were affixed to a 3D printed base to prevent shifting when moving the setup or putting live mosquitoes into the box. One side of the box was transparent to allow the camera to monitor the inside of the box, and the top of the box was sealed by another transparent plate. With this design, the light from the LED could penetrate the inside of the box. This was to eliminate shadows in the images captured by the camera and to prevent the characteristics of shadows from affecting neural network training.

We used a Raspberry Pi (Raspberry Pi three Model B, Raspberry Pi Foundation, United Kingdom) to control a DC motor (DC Gearbox Motor, TT, China) and the camera used to capture images (Figure 4A). The captured images were stored in a secure digital (SD) memory card in the Raspberry Pi. The Raspberry Pi and DC motor were housed in a 3D-printed protective shell to allow attachment and protect the circuits of the Raspberry Pi and DC motor (Figure 4B).

**FIGURE 5:**
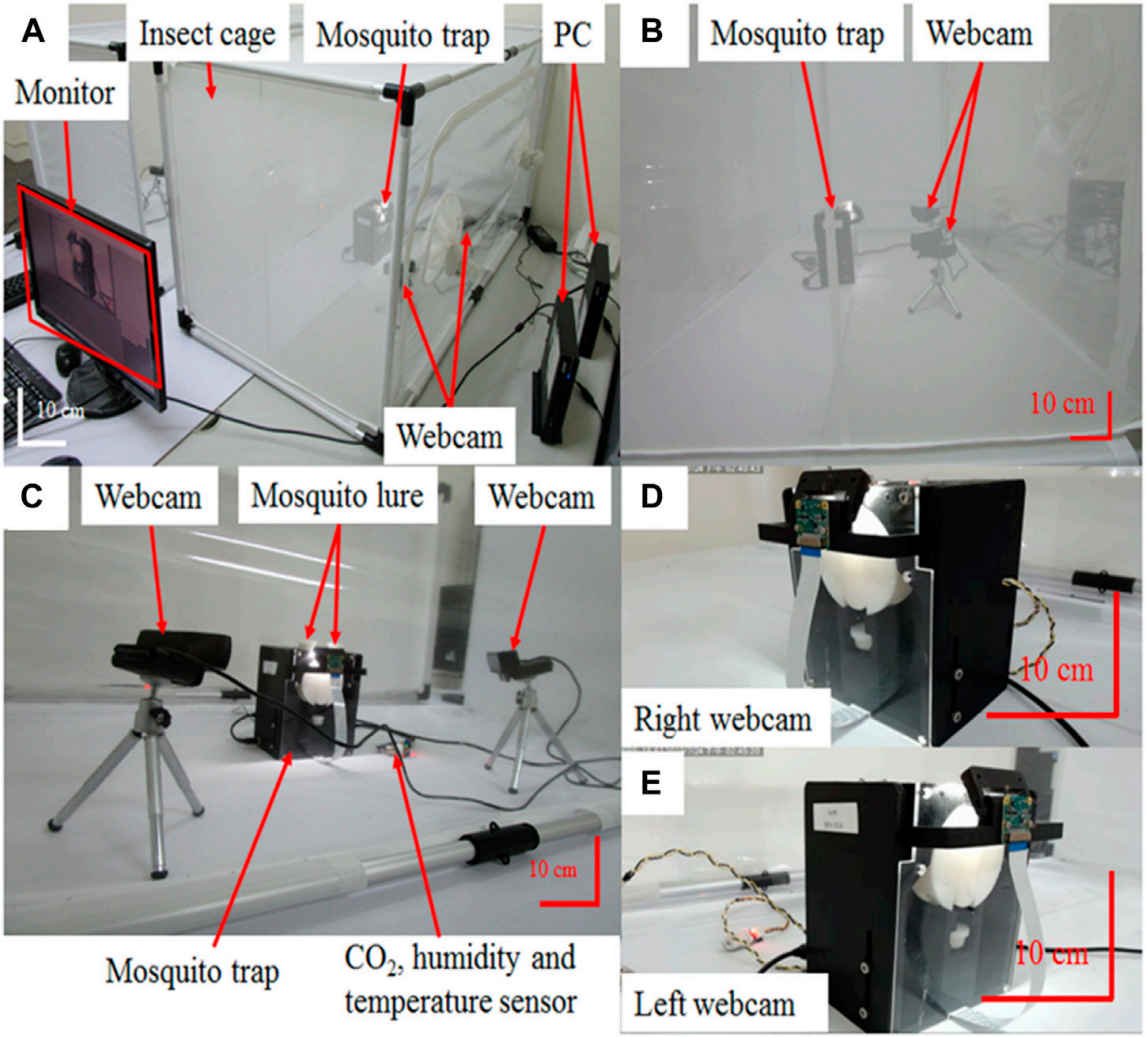
Performance test of the core device of the developed mosquito trap to identify and classify mosquitoes. **(A)** The mosquito trap set up for a live mosquito capture test. PCs and cameras are placed on the left and right sides of the mosquito trap, and the screen displays the captured image. **(B,C)** Mosquito trap and two cameras in the mosquito net. **(D,E)** Real-time image of the mosquito trap core device from the right (Supplementary Movie S7) and left (Supplementary Movie S8) sides of the mosquito trap. The camera recorded the statuses of mosquitoes and mosquito traps in flight. After each experiment, the mosquito data were recorded, and statistical data were used to calculate the capture rate and recognition rate of the experiment.

To obtain images of moving mosquitoes, we confined live mosquitoes to the box and used a rotating rod to disturb the entire system at a constant rate. The rotating rod produced seven knocks on the system for approximately 10 s, rested for 10 s, and repeated the cycle (Figure 4B; Supplementary Figure S5A and Supplementary Movie S2). This disturbance ensured that a mosquito would fly continuously inside the 3D printed box, as shown in Figure 5. The camera then captured an image similar to that captured by a mosquito trap when a live mosquito flies over the capture area (Figure 5B, Supplementary Movie S3). When the interference stopped, the mosquito landed on the box wall, and the captured image reflected the state at which the mosquito had landed after flying (Supplementary Figures S5C,D, Supplementary Movie S4, and Supplementary Movie S5).

We then fed the video feed into the previously mentioned motion detection algorithm to obtain subimages. Since the subimages sometimes included unusable images, they were manually classified based on mosquito presence or absence. Accordingly, we were able to quickly obtain valuable data.

### Training the recognition algorithm

To identify different kinds of mosquitoes, SqueezeNet was selected as our neural network model (Figure 3), and we further improved it by applying the transfer learning technique based on the mosquito datasets for both static specimens (Supplementary Figure S4) and live mosquitoes (Figures 4C, D), including *Ae. aegypti* and *Cx. quinquefasciatus*, that we obtained. The training, validation, and testing set splits are listed in Supplementary Table S1 and Supplementary Table S2. The confusion matrix of the testing set is listed in Table 1. We also tested the core device of the mosquito trap to identify and classify *Cx. quinquefasciatus* and *Ae. aegypti* mosquitoes (Supplementary Figure S6 and Supplementary Movie S6). Each test consisted of a manual feeding of 10 *Cx. quinquefasciatus* and 10 *Ae. aegypti* mosquitoes into the detection zone of the core device to observe how the mosquito trap behaved. Three of the six tests test used dead mosquitoes (Table 2, test 1–3), while the remaining three tests used live mosquitoes (Table 2, test 4–6).

**TABLE 1:** Confusion matrix. A mosquito image recognition system was developed using the SqueezeNet neural network, and transfer learning was applied using the mosquito datasets, which were generated by fixing a mosquito specimen on an insect pin and rotating it 360° along the *Z*-axis, while the camera rotated along the *X*-axis. LED brightness was adjusted to simulate mosquitoes under different sunlight and other light source conditions. The training database contained images of mosquitoes of different species, sexes, and ages as well as images with different brightness levels and taken at different angles. Through the live mosquito dynamic image capturing device, pictures of mosquitoes in flight and in other positions were taken, increasing the diversity of the training data.

Predicted class				
		Ae. aegypti (%)	Cx. quinquefasciatus (%)	Empty (%)
True Class	Ae. aegypti	91.57	1.43	6.99
	Cx. quinquefasciatus	1.25	89.29	9.45
	Empty	4.89	4.56	90.54

**TABLE 2:** Test results of the mosquito trap (mosquito trap accuracy represents the percentage of mosquitoes captured to the intended chamber decided by the algorithm). Static mosquito images taken from various angles, at different light intensities and with different shadows were entered into the dataset, as well images of live mosquitoes in flight and in other movement positions. These images were entered into the SqueezeNet neural network for training. Models that can recognize mosquito species were generated. When a mosquito enters the mosquito trap area, the smart mosquito trap image recognition unit recognizes the mosquito type and communicates with the mosquito trap structure to quickly force the mosquito into the corresponding chamber. The whole process can be completed within 0.1–0.3 s so that mosquitoes will not escape. In this table, both live mosquitoes and dead mosquitoes were added to the mosquito capture area, and the smart mosquito trap capture status was recorded. Each test was performed independently and repeated 3 times.

Test number	State of mosquitoes	Algorithm accuracy	Mosquito trap accuracy
Test 1	Dead	90% (9/10)	100% (10/10)
Test 2	Dead	90% (9/10)	100% (10/10)
Test 3	Dead	70% (7/10)	100% (10/10)
Test 4	Alive	100% (10/10)	80% (8/10)
Test 5	Alive	100% (10/10)	90% (9/10)
Test 6	Alive	100% (10/10)	100% (10/10)

### Capture efficiency of the smart mosquito trap

After confirming that the recognition result was correct, the core device was placed in an insect cage (60 cm × 60 cm × 120 cm; BD6S620, MegaView Science Co. Ltd., Taiwan), and the mosquito lure was used to test live mosquito trapping efficiency. An air-conditioning system kept the environment at a constant temperature (27°C), and the mosquito trap and two cameras (C920E, Logitech Co., Ltd., Taiwan) were placed in the center of the insect cage, as shown in Figure 5A. The two cameras were placed on the left and right sides of the mosquito trap (Figures 5B, C). Then, the zipper of the insect cage was closed, and the camera transmission line and power cord were fed through. Before releasing the mosquitoes, the mosquito trap was activated and the cameras were turned on. We confirmed that the recording screen could be visualized properly and that the recorded images could be stored on the computer’s hard disk (Figure 5A). After turning on the recording device, 50 mosquitoes were released into the insect cage through open cuffs, which were then closed once the mosquitoes entered the cage. Mosquitoes were able to fly freely in the narrow space and were caught in the mosquito trap. The camera recorded the mosquitoes’ flight status and mosquito trap status and classified the images as right video images (Figure 5D, Supplementary Movie S7) and left video images (Figure 5E, Supplementary Movie S8). After each capture, capture data were recorded, and the capture rate and recognition rate were calculated based on statistical data. In addition, a smart mosquito trap integrated with a BG trap was placed in the insect cage (with an air conditioning system room, Supplementary Figure S7) and the small living room of a simulated house (3.1 m × 2.3 m × 3 m, without furniture and air conditioning, Supplementary Figure S8) to perform the abovementioned capture rate and recognition rate tests.

### Statistical analysis

Statistical analysis was performed using GraphPad Prism software (version 5.0). Use standard errors for statistical validation when testing catch and recognition rate. We analyzed the lure or temperature for capture and recognition rates using Mann‒Whitney-Wilcoxon paired t tests. A *p*-value less than 0.05 was considered statistically significant.

## Results

### Mosquito capture experiment and recognition accuracy

**FIGURE 6:**
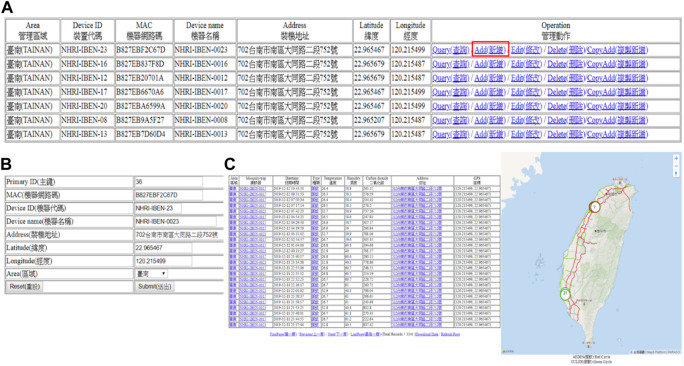
Data visualization and the cloud data collection platform (http://mosquitotrap.nhri.org.tw/). **(A)** Use the mosquito trap website to query, add, edit, delete, and manage the data of each mosquito trap. If a new mosquito trap needs to be added to the online system, click “Add” on the website. **(B)** For the mosquito trap to be added, fill in the machine’s media access control (MAC) address, mosquito trap code, name, address, latitude and longitude, and area and click submit. **(C)** After adding a mosquito trap, the mosquito trap website will be updated. The table on the left side and the map on the right side display the information uploaded by the newly added mosquito trap, such as the name of the mosquito trap; the types of mosquitoes caught; the local temperature, humidity, and CO_2_ concentration; and capture information such as mosquito location and GPS latitude and longitude.

Supplementary Figure S6 and the associated recorded video from the experiment (Supplementary Movie S6) show the mosquito trap core device used to identify and classify *Cx. quinquefasciatus* (left container) and *Ae. aegypti* (right container) mosquitoes. The mosquito is manually herded to the detection zone of the core device directly from the top, as indicated by the red arrow in Supplementary Figure S6A. A mosquito flying into the detection zone activates the system; then, the capture plate rotates and drives the mosquito into the proper storage container. For example, in this study, *Cx. quinquefasciatus* and *Ae. aegypti* were captured in the left and right collection containers, respectively (Figure 6). According to our experimental data, mosquitoes constantly approached the trap during its operation, and the trap captured the mosquitoes successfully.

Regarding the recognition accuracy, SqueezeNet was applied to 1) train a model that can recognize mosquito types (Supplementary Figure S3 and Supplementary Movie S1) and 2) test the function of mosquito recognition under static and live mosquito conditions (Supplementary Table S1 and Supplementary Table S2). Our experimental data indicated that the real-time recognition accuracy rates were more than 91.57% and 89.29% in the test set images (Table 1) and live *Cx. quinquefasciatus* and *Ae. aegypti* system (Supplementary Figures S5C,D), respectively.

### Examination of the developed mosquito trap with artificial intelligence (AI)-based mosquito image detection

The intermediate deep neural networks required for the procedure introduced in the above section were developed and trained. First, the AlexNet classifier network for the mosquito contour classification shown in Figure 3 was trained and examined. The data reported that the developed neural network achieved approximately 91.57% accuracy in predicting the test set images (Table 1). Our system performed real-time classification of live *Cx. quinquefasciatus* or *Ae. aegypti* mosquitoes that were manually fed into the detection zone of the core device (Supplementary Figure S6 and Supplementary Movie S6). When integrated into a mosquito trap, our capture system can be used to successfully distinguish *Cx. quinquefasciatus* (Supplementary Figures S6A–C) and *Ae. aegypti* (Supplementary Figures S6D–F) mosquitoes *in vivo* and sorted into the proper chamber after identification.

To further test our mosquito trap system, we conduct the tests shown in Table 2. In these tests, the overall accuracy of mosquito recognition was 95 ± 7.6% (57/60), and the capture mechanism had a success rate of 90 ± 8.2% (27/30) in the tests with live mosquitoes. The capture and identification tests involving the core device of the prototype trap used real-time capture and classification video data to determine the effectiveness of the mosquito trap. The camera fully recorded the statuses of the mosquitoes and mosquito traps. Approximately ten experiments revealed that the accuracy of mosquito classification was more than 92 ± 3.4%. Although the capture rate was not very good, it was still nearly 44 ± 9.6% (Figure 5; Table 3). We also found that the number of lures seemed to have a slight effect on the average catch number, but the difference was not statistically significant (3 vs. 1 lure, 48% vs. 39%, *p* = 2.79, Mann-Whitney test).

**TABLE 3:** Performance test results of the core device of the mosquito trap in the insect cage. The test setup is shown in Figure 5. Fifty *Ae. aegypti* mosquitoes were placed into the insect cage. The mosquitoes were allowed to fly freely in the limited space and were captured and identified by the mosquito trap. The size of the tested insect cage was 60 cm × 60 cm × 120 cm, and the test environment temperature was maintained at 27°C ± 1°C by an air conditioning system. Each test was performed independently and repeated 10 times.

Experiment number	Time	Capture rate (%)	Recognition rate (%)	Number of Ae. aegypti released	Number of captures	Recognition times	Recognition of Ae. aegypti	Recognition of Cx. quinquefasciatus	Number of lures
Experiment 1	15:49–10:00	42	87	50	21	39	34	5	3
Experiment 2	15:30–09:30	42	86	50	21	37	32	5	3
Experiment 3	15:30–11:30	66	93	50	33	44	41	3	3
Experiment 4	15:30–12:05	54	97	50	27	34	33	1	3
Experiment 5	16:20–13:00	38	94	50	19	32	30	2	3
Experiment 6	15:30–11:30	46	97	50	23	29	28	1	1
Experiment 7	15:30–11:30	32	92	50	16	24	22	2	1
Experiment 8	15:30–11:30	42	92	50	21	38	35	3	1
Experiment 9	15:30–11:50	42	91	50	21	35	32	3	1
Experiment 10	15:30–11:30	32	91	50	16	32	29	3	1

### Efficacy of the modified BG trap integrated with the smart mosquito trap

Based on the good catch rate of the BG trap (Johnson et al., 2012; Barrera et al., 2013; Li et al., 2016), we integrated the core device of the mosquito trap into a commercial BG trap to form a new type of smart mosquito trap and then conducted tests related to mosquito trapping (Supplementary Figure S7). In a laboratory where the temperature was controlled at 27°C ± 1°C, the newly integrated smart mosquito trap was placed in a small insect cage for testing. The results showed that the average catch rate and recognition rate increased by 67 ± 25.0% and 97 ± 2.9%, respectively (Table 4). Afterward, we expanded the space in which the mosquitoes could fly by 50-fold by placing the trap in a simulated living room without air conditioning (fluctuations of 27–33°C) and conducted additional mosquito trap tests (Supplementary Figure S8). Similar results were obtained, with an average capture rate of 49 ± 17.7%; the recognition rate reached 90 ± 8.3% (Supplementary Table S3).

**TABLE 4:** Experimental results of the BG trap integrated with the developed mosquito trap core device in an insect cage. The test setup is shown in Supplementary Figure S7. Fifty *Ae. aegypti* mosquitoes were placed into the insect cage, which was 60 cm × 60 cm x 120 cm, for capture and identification by the mosquito trap. The test environment temperature was maintained at 27°C ± 1°C by an air conditioning system. Each test was performed independently and repeated 6 times.

Experiment number	Time	Capture rate (%)	Recognition rate (%)	Number of Ae. aegypti released	Number of captures	Recognition times	Recognition of Ae. aegypti	Recognition of Cx. quinquefasciatus	Number of lures
Experiment 1	16:10–11:30	64	100	50	32	38	38	0	1
Experiment 2	14:20–11:00	80	100	50	40	46	46	0	2
Experiment 3	15:30–11:00	86	100	50	43	50	50	0	2
Experiment 4	14:20–11:00	96	94	50	48	47	44	3	2
Experiment 5	15:00–11:00	54	97	50	27	38	37	1	2
Experiment 6	15:00–11:00	20	93	50	10	15	14	1	2

### Data visualization and the cloud data collection platform

The user connects to the project website (Figure 6), and all the data are transmitted to the user on the backend, including the area where the mosquitoes are caught (Figures 6A, C), the mosquito trap number, address, GPS coordinates (Figure 6B), date and time, type of mosquito vector, temperature, humidity, *etc.* The user accesses the data *via* a client web page; weather data are constantly pulled from the Central Weather Bureau (CWB) *via* an open application programming interface (API) connection.

## Discussion

To the best of our knowledge, this is the first study that used a CNN to extract features from images of adult mosquitoes to identify *Cx. quinquefasciatus* and *Ae. aegypti* mosquitoes and capture them safely in real time. The CNN was able to distinguish between *Cx. quinquefasciatus* and *Ae. aegypti* test set images, with an accuracy rate of 92%. The smart trap also uses AI algorithms to identify whether the insect entering the trap is a vector mosquito of interest. The trap has the advantage of operating with low power consumption and is made using inexpensive 3D printing, making it a relatively inexpensive tool for mosquito control. The trap design is suitable for high-throughput manufacturing and utilizes commercially available mosquito attractants.

Traditional ventilation mosquito traps have the disadvantages of high-power consumption and a low capture rate. Therefore, in this study, we proposed a new method for catching live mosquitoes (Figure 2; Supplementary Figure S2). When the mosquito trap is inactive, the slide rail of the catch block is aligned with the opening of the cover. The chassis on the slide rail forms a space between the lowest point and the cover hole on the slide rail, forming the capture area. When a mosquito enters this space, the motor of the capture block activates to rotate the capture block. This action immediately closes the opening and the gap, pushing the mosquito into the capture space. When the capture block rotates 90°, the chassis on the slide rail compresses the space due to gravity and forces the mosquito to exit the capture block. The chassis will eventually reach the top of the slide rail. At this time, the capture space is completely compressed, forcing the mosquito into the collection box. After the collection is complete, the capture block will rotate in the opposite direction back to its original position, and the chassis on the slide rail will return to the bottom due to gravity, opening up the capture space to catch the next mosquito. With the proposed rotation method, the mosquito trap can effectively capture target mosquitoes. The design of the mechanism can effectively force the captured mosquitoes into the storage area (Supplementary Figure S6). Our experimental data indicate that the trap design is feasible and will not harm mosquitoes (Table 2). We achieved a 90% capture rate in a laboratory setting.

The collection of mosquito specimens is an essential part of neural network training. Therefore, it is necessary to design an ideal imaging system to photograph static mosquito specimens (Supplementary Figure S3 and Supplementary Figure S4). The training results from SqueezeNet can be applied to only static mosquito specimens, and it might not be feasible to classify live mosquitoes. Accordingly, a different camera system was designed to specifically target live mosquitoes (Figures 4A,B). The images of live mosquitoes are closer to reality than those of static mosquitoes, as live mosquitoes fly and move, unlike static mosquito specimens (Figures 4C, D). However, mosquitoes do not change their posture often, so an interference system was also designed. To obtain images of moving mosquitoes, we confined live mosquitoes to a box and used a rotating rod to disturb the entire system at a constant rate. The rotating rod produced seven knocks on the system for approximately 10 s, rested for 10 s, and then repeated the cycle (Figures 4A, B). The best and most convenient motor tapping system design was obtained (Supplementary Figures S5A,B), and based on this, many images of living mosquitoes in different flight positions were taken (Supplementary Figures S5C,D). The developed mosquito trap has an excellent recognition effect due to not only the use of the SqueezeNet neural network for training but also the content of the database; for example, we used both static and live mosquito images for training for the first time.

The test results were similar to what we expected and suggested that building a smart mosquito trap using our design is practical. The system, however, calls for some improvements that could be made in the future. First, the neural network’s accuracy could certainly be further improved; additionally, we trained the network on only two different types of mosquitoes, namely, *Cx. quinquefasciatus* and *Ae. aegypti*. In real-world settings, one would anticipate that other types of insects would enter the capture area at some point. We thus need to obtain a more comprehensive dataset on all possible insects that may enter the capture area and train our neural network to recognize these insects so that our mosquito trap can capture only the target species, namely, *Ae. aegypti* mosquitoes. Second, our capture plate successfully forced live mosquitoes into the intended chamber approximately 90% of the time. In the failed cases, we found that the mosquitoes got stuck between the capture plate and the ceiling or were forced into the incorrect chamber by rotation of the capture plate. Future modification of the mosquito trap should address this problem to make the capture process more effective. The next step in our mosquito trap research is to deploy the trap in a more general experimental setting to elucidate effective ways to capture mosquitoes in the wild.

In addition, the BG trap is currently considered the “gold standard” for urban *Aedes* mosquito capture (Johnson et al., 2018). Even so, a BG trap lacks a real-time capture and classification video function to detect the types of mosquitoes caught by the mosquito trap. The smart mosquito trap we developed has been modified to include this important function. The smart mosquito trap can automatically distinguish between *Aedes* and non-Aedes mosquitoes when they enter the trap, count them, and wirelessly transmit the results to the cloud server in real time. Vector control professionals can establish the data density and accuracy of monitoring procedures for vector mosquitoes to understand the adult density index and population dynamics. Although a second-generation BG-Counter two trap has been developed and sold recently, its price may still be higher than that of our smart mosquito trap. The smart mosquito trap we have developed can provide valuable functions at a low cost so that effective and affordable mosquito surveillance systems can be widely implemented.

Commercially available traps usually combine a specific sense of smell and vision to attract mosquitoes. These lures are usually used in combination and often play an important role. In our experiments, although the trapping rate reached nearly 50% (Table 3 and Table 4), if the capture rate could be increased, the control of vector mosquitoes could be improved. Adjusting the lures could be a method to increase the catch rate of the trap. In addition to CO_2_ and chemistry (simulating the body similar to the function of attracting mosquitoes), providing additional stimuli, such as light or sound, may improve the capture efficiency. For several mosquitoes, low-wavelength light (green to blue) is very attractive (Burkett et al., 1998; Mwanga et al., 2019). Increasing the light intensity will also affect the attractiveness to mosquitoes. Sound is also a way to lure mosquitoes. Recent studies have indicated that using 450- or 500-Hz sound as a bait can catch mosquitoes (Staunton et al., 2021). Therefore, applying different forms of lures to the trap will greatly improve the efficiency of the developed smart mosquito trap for catching mosquitoes.

Recent studies have shown that climate influences the capture rate of mosquito traps, which in turn affects the efficiency of traps (Crepeau et al., 2013). Taiwan has a subtropical climate, and the most common area for dengue fever in Taiwan is the south. The temperature mostly remains above 30°C, which is suitable for mosquito breeding. Therefore, we used 30°C as the ambient temperature cutoff to detect whether temperature had an impact on the mosquito trap efficacy. Ambient temperature had no effect on the capture rate (*p* = 0.64, Mann-Whitney test) or identification rate (*p* = 0.39, Mann-Whitney test) of the mosquito trap (Supplementary Table S3). We also found that the mosquitoes caught were still alive and that their external morphological characteristics were not obviously destroyed. Therefore, identification ability was improved; moreover, live mosquitoes are beneficial in analyzing whether they harbor dengue virus or other viruses. Taken together, the modified BG trap integrated with the developed smart mosquito trap had good effects in capturing and identifying mosquitoes, which will help control mosquito vectors.

Currently, all commercially available mosquito traps lack effective environmental parameter detection mechanisms, big data cloud platform connections, and mosquito identification and capture mechanisms (Schatz et al., 2010). Therefore, various environmental sensors, such as CO_2_, temperature, and humidity sensors, are integrated into the new smart mosquito trap. The traps also connect to one another *via* the Internet of Things (IoT) (Liao et al., 2019); the traps collect and record environmental data in high-risk areas, and IoT technology and big data cloud analysis can be used to predict the next area at high risk of dengue fever. This allows the implementation of environmental epidemic prevention and control strategies as soon as possible. However, the related vector mosquito identification system should be improved; deep learning technology should be used in the development of image identification systems to track dynamic ranges (Kim et al., 2019), species, and distribution densities of vector mosquitoes to identify potential epidemic areas at high risk. The prevention of mosquito-borne diseases should be carried out in one step.

The environmental data detected by the new mosquito trap in this study have been uploaded to an open data platform (Figure 6) (http://mosquitotrap.nhri.org.tw/), not only for data visualization but also to allow developers to develop other applications to extend the results of this study. The collected data can also be used as a key dataset for deep learning algorithms for the prediction of mosquito epidemic situations in the future. Integrating additional information, such as ultraviolet ray intensity and rain probability, will allow people to monitor their environment and the status of vector mosquito-borne diseases at any time from any location, similar to weather forecasts. The collected data will also allow further academic research. With contributions from makers and the use of folk technologies, this type of intelligent mosquito trap can be utilized worldwide in locations such as schools, public places, and even private residences, increasing popularization and improving the control of future epidemics.

## Conclusion

In summary, we developed a smart mosquito trap system that correctly differentiated between *Cx. quinquefasciatus* and *Ae. aegypti* mosquitoes and captured them in different chambers with an accuracy of approximately 92%. The core prototype achieved a capture rate of up to 90% under laboratory testing conditions. In combination with a BG trap, the recognition rate and capture rate were 90% and 49%, respectively, when tested in a simulated living room setting. These results, along with the humidity, temperature, and CO_2_ concentration, can be instantly updated to the cloud when a mosquito is captured. This smart mosquito trap can help control mosquito-borne diseases and predict their possible outbreaks. We expect that this system could be used in indoor urban areas and at rainfall collection facilities in tropical regions in developing countries. We have proven that such a mosquito trap is practicable by improving the real-time recognition algorithm and mechanical design. The deployment of this type of mosquito trap in a real-world setting to help prevent mosquito-borne diseases is within sight. We hope that in the future, the model can be embedded in a mobile application (app) to allow for community participation and thereby facilitate efforts to control vector-borne diseases. Indeed, this model can improve vector control operations through fast and reliable identification of target species and provide insights into their biology and ecology.

## Data Availability

The original contributions presented in the study are included in the article/Supplementary Material, further inquiries can be directed to the corresponding authors.

## Supplementary material

Supplementary Table S1 The number of images for each type of mosquito used in the training set.

Supplementary Table S2 The number of images used in the validation set and the testing set.

Supplementary Table S3 Experimental results of the integration of the BG trap and the developed core device of the mosquito trap in a small, simulated living room. The test setup is shown in Figure S8. Fifty *Ae. aegypti* mosquitoes were released into the living room, which was 3.1 m x 2.3 m x 3 m, for capture and identification by the mosquito trap. The mosquito trap contained 2 lures. The ambient temperature ranged from 28°C to 32°C during the period of trapping. Each test was performed independently and repeated 29 times.

Supplementary Figure S1 Hardware operation and signal transmission. The model trained by the neural network is loaded into the Raspberry Pi to recognize types of mosquitoes. The image captured by the camera is transmitted back to the Raspberry Pi for file processing, and the temperature, humidity, and CO_2_ concentration are recorded. The Raspberry Pi then prompts the capture mechanism to rotate the motor for capture and returns the turntable to its original position with a photodetector. Then, the data are uploaded to the cloud database for statistical analysis, and the captured mosquito can be viewed on a browser.

Supplementary Figure S2 Capture mechanics of the proposed mosquito trap. First, the capture area is opened for mosquitoes to enter. When a mosquito is detected, the capture area rotates, immediately blocking the path to the exit. When the plate turns 90º, the impact board is then lowered by gravity, forcing the mosquito into the storage chamber. After the mosquito has entered in the storage area, the capture mechanism returns to its original state. When turning, the impact board stays at the bottom, preventing the mosquito from escaping. Gravity then pulls the impact board back down, and the mosquito trap returns to its normal state.

Supplementary Figure S3 Mosquito image collection and database establishment. **(A)** The MATLAB GUI interface for the static mosquito shooting device (Movie S1). **(B)** The design of the mosquito image collection device. **(C)** The camera rotates vertically to take pictures of mosquitoes. **(D)** When shooting mosquitoes, the camera is rotated on a horizontal plane.

Supplementary Figure S4 Static mosquito images at different viewing angles from our image collection system. First, we fixed the mosquito onto an insect pin and rotated it 360 degrees along the Z-axis. Simultaneously, the camera rotated along the X-axis and adjusted the brightness with an LED to simulate images of mosquitoes under different sunlight and light source conditions. The training database contained mosquitoes of different types, sexes, and ages, as well as images taken under different light conditions and at different angles.

Supplementary Figure S5 Overview of the dynamic mosquito studio and its operation, including the dynamic studio's internal perspective for mosquitoes. **(A)** The operation of the camera when a live mosquito is photographed (Movie S2). **(B)** A mosquito in the shooting box when the live mosquito camera was recording (Movie S3). **(C)** Recognition of *Cx. quinquefasciatus* after imaging and training by the neural network (Movie S4). **(D)** Recognition of *Ae. aegypti* after imaging and training by the neural network (Movie S5).

Supplementary Figure S6 Mosquito trap's core device identifies and classifies *Cx. quinquefasciatus* and *Ae. aegypti* (Movie S6). **(A)** A mosquito (*Cx. quinquefasciatus*) is manually fed into the trap from the top. **(B)** The capture plate rotates and pushes the mosquito into the storage chamber. **(C)** The mosquito is now trapped in the storage chamber. **(D)** A mosquito (*Ae. aegypti*) is manually fed into the trap from the top. **(E)** The capture plate rotates and pushes the mosquito into the storage chamber. **(F)** The mosquito is now trapped in the storage chamber.

Supplementary Figure S7 Performance test of the developed smart mosquito trap integrated into the commercial BG trap. **(A)** Combined BG trap and mosquito trap mechanism and airflow diagram. **(B)** Capture test involving the BG trap combined with the mosquito trap in an insect cage. The power cables were fed through the seal of the zipper. After the insect cage zipper was closed, the two 18-cm cuff openings were kept open for mosquitoes to enter. Before placing the mosquitoes in the cage, the mosquito trap as activated. After turning on the trap, 50 mosquitoes were put into the insect cage through the open sleeve. After placing the mosquitoes in the cage, the sleeves were closed tightly, trapping the mosquitoes in the insect cage. The mosquitoes were allowed to fly freely in the limited space and were caught by the mosquito traps in each experiment.

Supplementary Figure S8 Performance tests of smart mosquito traps integrated into BG traps in a small, simulated living room. **(A)** The room layout and location where the combined BG trap and smart mosquito trap was placed for capture testing. **(B,C)** Actual photos of the room and the actual placement of the BG trap. No furniture or air conditioning was placed in the room to allow maximum space for the mosquitoes; the window remained closed. After the mosquito trap was activated, 50 mosquitoes were released into the room. The mosquitoes were allowed to fly freely in the room, and the trap captured the mosquitoes. After releasing the mosquitoes into the room, the sleeves of the insect cage were closed. The mosquitoes were allowed to fly freely in the limited space and were caught by the trap in each experiment.

Supplementary Movie S1 The establishment of the mosquito image database.

Supplementary Movie S2 Dynamic mosquito image collection mechanism.

Supplementary Movie S3 The dynamic mosquito studio inside perspective view.

Supplementary Movie S4 Shoot and recognize the *Cx. quinquefasciatus* in the dynamic mosquito studio.

Supplementary Movie S5 Shoot and recognize the *Ae. aegypti* in the dynamic mosquito studio.

Supplementary Movie S6 The core device of the trap to identify and classify the mosquitoes.

Supplementary Movie S7 The camera recorded the mosquitoes' flight status and mosquito trap status and classified the images as right video images.

Supplementary Movie S8 The camera recorded the mosquitoes' flight status and mosquito trap status and classified the images as left video images.
