# Supplementary material for: An IoT-based smart mosquito trap system embedded with real-time mosquito image processing by neural networks for mosquito surveillance
Source: Front Bioeng Biotechnol. 2023 Jan 20;11:1100968. doi: 10.3389/fbioe.2023.1100968 (PMC9895108; doi:10.3389/fbioe.2023.1100968)
Supplement: Supplementary file 8 [file DataSheet1.docx]

**Supplementary Tables**

**Table S1.**

| **Type of mosquito** | **Live images** | **Specimen images** |
| --- | --- | --- |
| ***Ae.*** ***aegypti*** | **56,018** | **28,800** |
| ***Cx. quinquefasciatus*** | **44,936** | **28,800** |
| **Empty** | **22,393** | **0** |

**Table S2.**

|  | ***Ae.* *aegypti***  **(Live)** | ***Cx. quinquefasciatus*** **(Live)** | **Empty** |
| --- | --- | --- | --- |
| **Validation set** | **10,592** | **14,625** | **2,965** |
| **Testing set** | **18,194** | **24,602** | **5,126** |

**Table S3.**

| **Experiment number** | **Time** | **Capture rate** | **Recognition rate** | **Number of *Ae. aegypti* released** | **Number of captures** | **Recognition times** | **Recognition of**  ***Ae. aegypti*** | **Recognition of**  ***Cx. quinquefasciatus*** | **Temperature** |
| --- | --- | --- | --- | --- | --- | --- | --- | --- | --- |
| **Experiment 1** | **15:30~12:20** | **30%** | **84%** | **50** | **15** | **31** | **26** | **5** | **28.3±1°C** |
| **Experiment 2** | **15:30~11:30** | **30%** | **83%** | **50** | **15** | **23** | **19** | **4** | **31.6±1°C** |
| **Experiment 3** | **16:00~12:00** | **36%** | **83%** | **50** | **18** | **24** | **20** | **4** | **29.6±1°C** |
| **Experiment 4** | **15:30~11:00** | **34%** | **80%** | **50** | **17** | **25** | **20** | **5** | **30.5±1°C** |
| **Experiment 5** | **14:50~10:50** | **62%** | **100%** | **50** | **31** | **22** | **22** | **0** | **30.1±1°C** |
| **Experiment 6** | **16:00~12:00** | **48%** | **100%** | **50** | **24** | **17** | **17** | **0** | **32.3±1°C** |
| **Experiment 7** | **15:00~11:00** | **76%** | **100%** | **50** | **38** | **36** | **36** | **2** | **31.3±1°C** |
| **Experiment 8** | **16:20~13:00** | **42%** | **87%** | **50** | **21** | **39** | **34** | **5** | **30.3±1°C** |
| **Experiment 9** | **15:30~ 11:30** | **42%** | **87%** | **50** | **21** | **37** | **32** | **5** | **32.3±1°C** |
| **Experiment 10** | **15:30~11:30** | **66%** | **93%** | **50** | **33** | **44** | **41** | **3** | **29.7±1°C** |
| **Experiment 11** | **15:30~11:30** | **54%** | **97%** | **50** | **27** | **34** | **33** | **1** | **28.9±1°C** |
| **Experiment 12** | **15:30~11:30** | **46%** | **82%** | **50** | **23** | **34** | **28** | **6** | **31.2±1°C** |
| **Experiment 13** | **15:30~11:30** | **50%** | **82%** | **50** | **25** | **39** | **32** | **7** | **29.8±1°C** |
| **Experiment 14** | **15:30~11:30** | **38%** | **94%** | **50** | **19** | **32** | **30** | **2** | **30.4±1°C** |
| **Experiment 15** | **15:30~11:30** | **32%** | **92%** | **50** | **16** | **24** | **22** | **2** | **32.6±1°C** |
| **Experiment 16** | **15:30~11:30** | **46%** | **97%** | **50** | **23** | **29** | **28** | **1** | **32.8±1°C** |
| **Experiment 17** | **15:30~11:30** | **42%** | **92%** | **50** | **21** | **38** | **35** | **3** | **30.9±1°C** |
| **Experiment 18** | **15:30~11:30** | **36%** | **85%** | **50** | **18** | **33** | **28** | **5** | **29.7±1°C** |
| **Experiment 19** | **16:10~11:30** | **30%** | **89%** | **50** | **15** | **37** | **33** | **4** | **31.2±1°C** |
| **Experiment 20** | **14:20~11:00** | **60%** | **79%** | **50** | **30** | **33** | **26** | **7** | **30.4±1°C** |
| **Experiment 21** | **14:20~11:00** | **40%** | **72%** | **50** | **20** | **25** | **18** | **7** | **31.3±1°C** |
| **Experiment 22** | **15:30~11:00** | **42%** | **91%** | **50** | **21** | **35** | **32** | **3** | **30.5±1°C** |
| **Experiment 23** | **15:30~11:00** | **32%** | **71%** | **50** | **16** | **24** | **17** | **7** | **29.2±1°C** |
| **Experiment 24** | **14:20~11:00** | **32%** | **91%** | **50** | **16** | **32** | **29** | **3** | **30.8±1°C** |
| **Experiment 25** | **14:20~11:00** | **64%** | **100%** | **50** | **32** | **38** | **38** | **0** | **30.3±1°C** |
| **Experiment 26** | **15:00~11:00** | **80%** | **100%** | **50** | **40** | **46** | **46** | **0** | **30.5±1°C** |
| **Experiment 27** | **15:00~11:00** | **86%** | **100%** | **50** | **43** | **50** | **50** | **0** | **28.9±1°C** |
| **Experiment 28** | **15:00~11:00** | **96%** | **92%** | **50** | **48** | **47** | **43** | **4** | **29.7±1°C** |
| **Experiment 29** | **14:50~10:50** | **54%** | **97%** | **50** | **27** | **38** | **37** | **1** | **30.5±1°C** |

**Supplementary Figures**


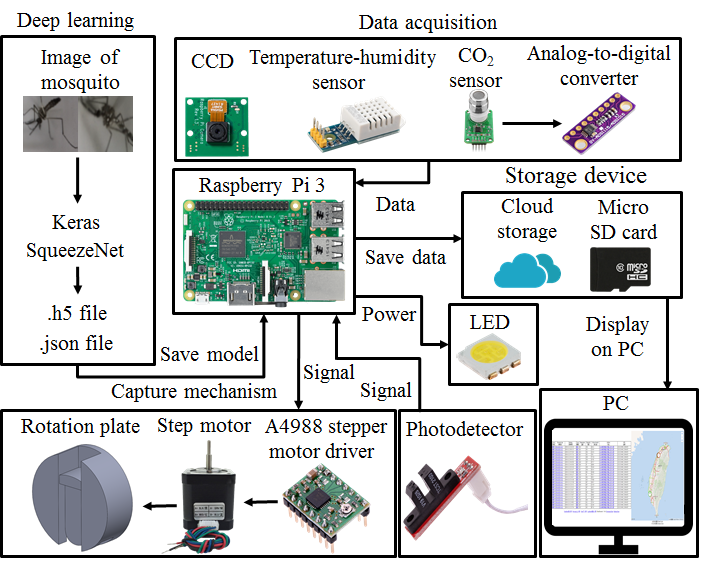


**Figure S1. Hardware operation and signal transmission.** The model trained by the neural network is loaded into the Raspberry Pi to recognize types of mosquitoes. The image captured by the camera is transmitted back to the Raspberry Pi for file processing, and the temperature, humidity, and CO_2_ concentration are recorded. The Raspberry Pi then prompts the capture mechanism to rotate the motor for capture and returns the turntable to its original position with a photodetector. Then, the data are uploaded to the cloud database for statistical analysis, and the captured mosquito can be viewed on a browser.


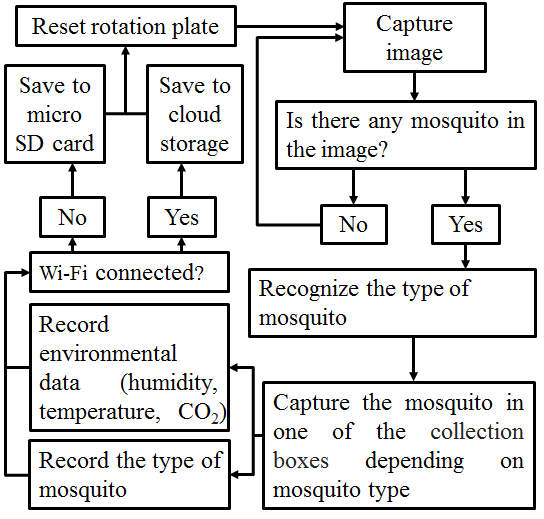


**Figure S2. Capture mechanics of the proposed mosquito trap.** First, the capture area is opened for mosquitoes to enter. When a mosquito is detected, the capture area rotates, immediately blocking the path to the exit. When the plate turns 90º, the impact board is then lowered by gravity, forcing the mosquito into the storage chamber. After the mosquito has entered in the storage area, the capture mechanism returns to its original state. When turning, the impact board stays at the bottom, preventing the mosquito from escaping. Gravity then pulls the impact board back down, and the mosquito trap returns to its normal state.


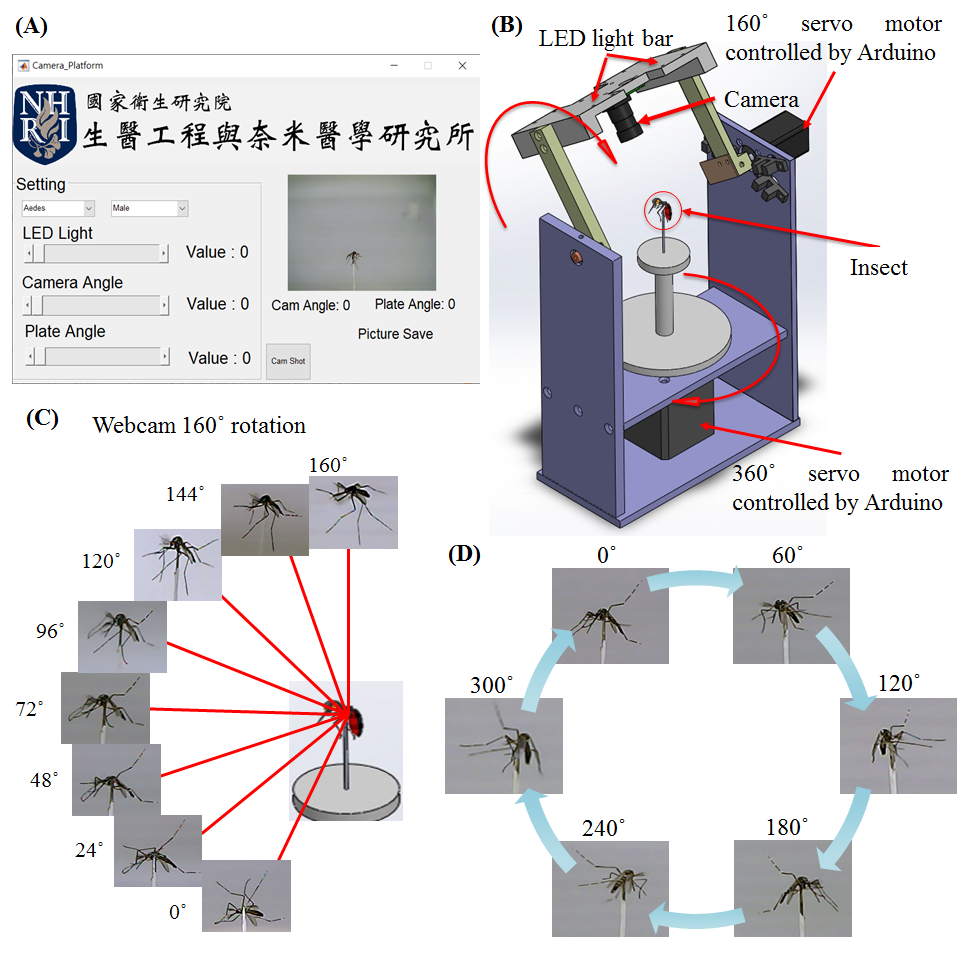


**Figure S3. Mosquito image collection and database establishment.** (**A**) The MATLAB GUI interface for the static mosquito shooting device (Movie S1). (**B**) The design of the mosquito image collection device. (**C**) The camera rotates vertically to take pictures of mosquitoes. (**D**) When shooting mosquitoes, the camera is rotated on a horizontal plane.


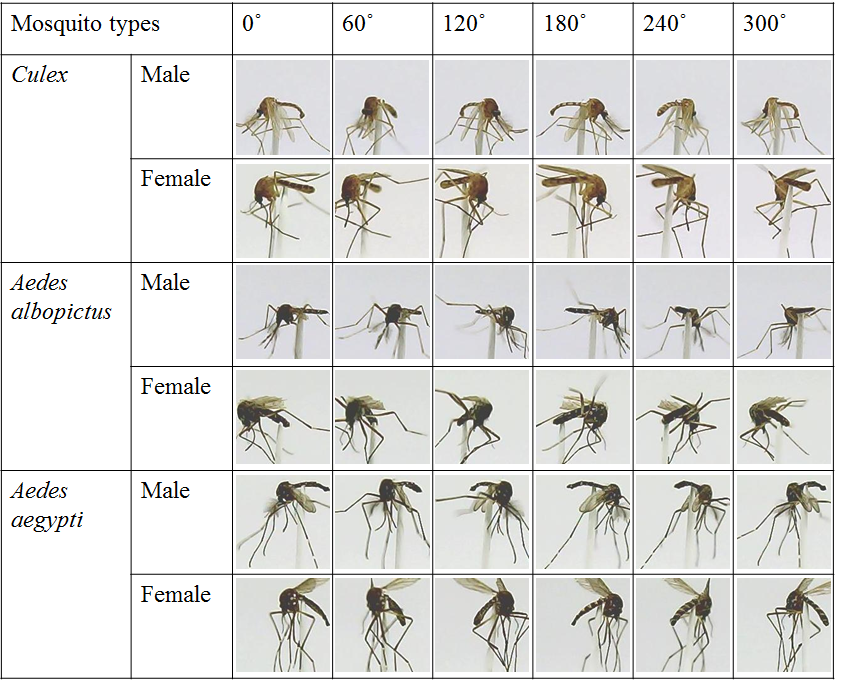


**Figure S4. Static mosquito images at different viewing angles from our image collection system.** First, we fixed the mosquito onto an insect pin and rotated it 360 degrees along the Z-axis. Simultaneously, the camera rotated along the X-axis and adjusted the brightness with an LED to simulate images of mosquitoes under different sunlight and light source conditions. The training database contained mosquitoes of different types, sexes, and ages, as well as images taken under different light conditions and at different angles.


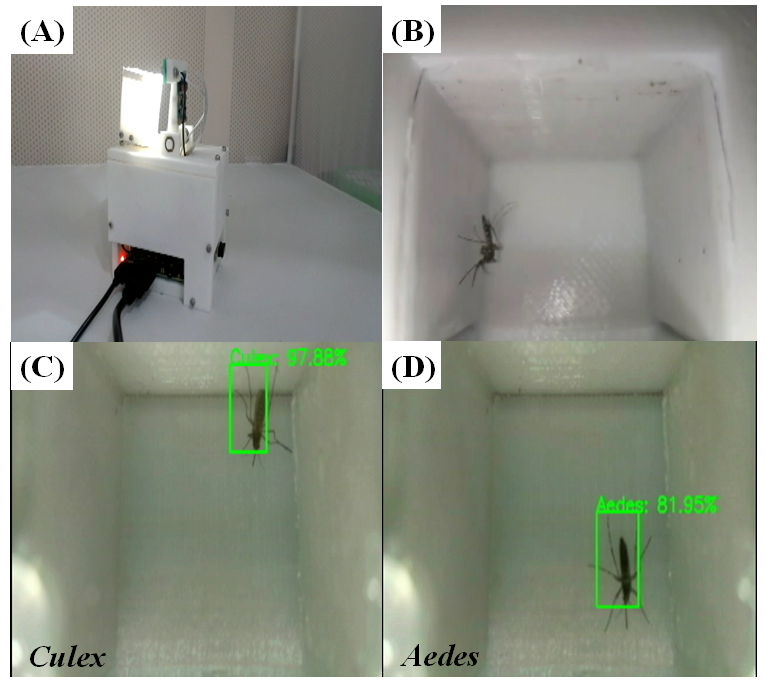


**Figure S5. Overview of the dynamic mosquito studio and its operation, including the dynamic studio's internal perspective for mosquitoes.** (**A**) The operation of the camera when a live mosquito is photographed (Movie S2). (**B**) A mosquito in the shooting box when the live mosquito camera was recording (Movie S3). (**C**) Recognition of *Cx. quinquefasciatus* after imaging and training by the neural network (Movie S4). (**D**) Recognition of *Ae. aegypti* after imaging and training by the neural network (Movie S5).


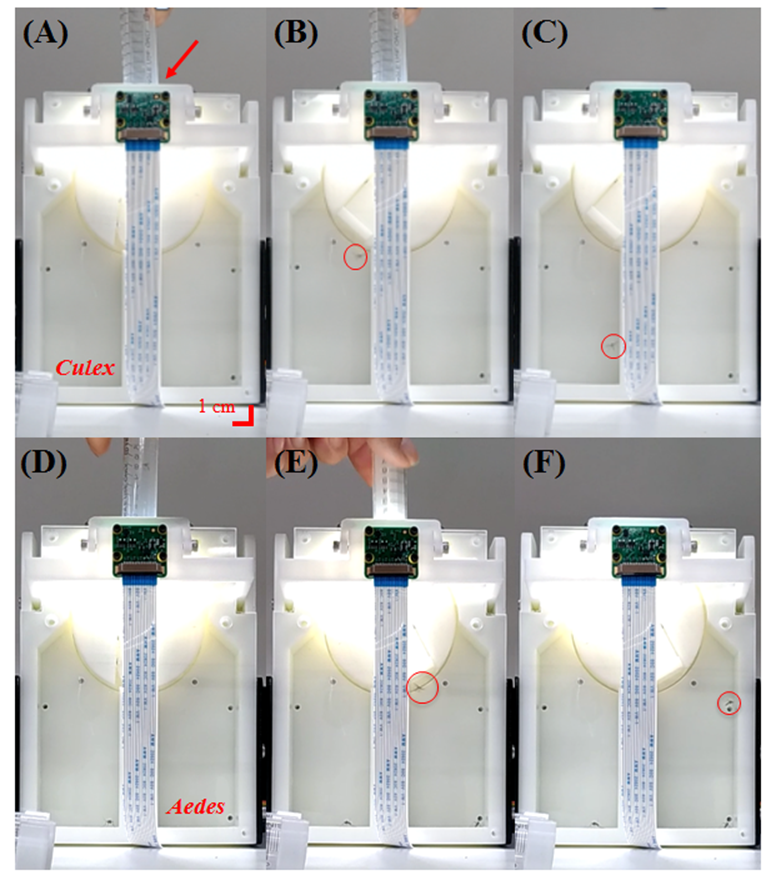


**Figure S6. Mosquito trap's core device identifies and classifies *Cx. quinquefasciatus* and *Ae. aegypti*** (Movie S6). (**A**) A mosquito (*Cx. quinquefasciatus*) is manually fed into the trap from the top. (**B**) The capture plate rotates and pushes the mosquito into the storage chamber. (**C**) The mosquito is now trapped in the storage chamber. (**D**) A mosquito (*Ae. aegypti*) is manually fed into the trap from the top. (**E**) The capture plate rotates and pushes the mosquito into the storage chamber. (**F**) The mosquito is now trapped in the storage chamber.


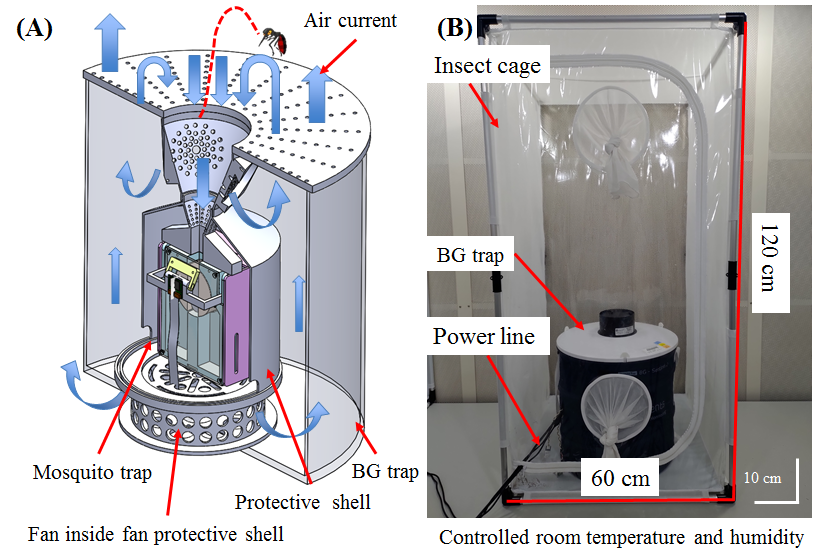


**Figure S7. Performance test of the developed smart mosquito trap integrated into the commercial BG trap.** (**A**) Combined BG trap and mosquito trap mechanism and airflow diagram. (**B**) Capture test involving the BG trap combined with the mosquito trap in an insect cage. The power cables were fed through the seal of the zipper. After the insect cage zipper was closed, the two 18-cm cuff openings were kept open for mosquitoes to enter. Before placing the mosquitoes in the cage, the mosquito trap as activated. After turning on the trap, 50 mosquitoes were put into the insect cage through the open sleeve. After placing the mosquitoes in the cage, the sleeves were closed tightly, trapping the mosquitoes in the insect cage. The mosquitoes were allowed to fly freely in the limited space and were caught by the mosquito traps in each experiment.


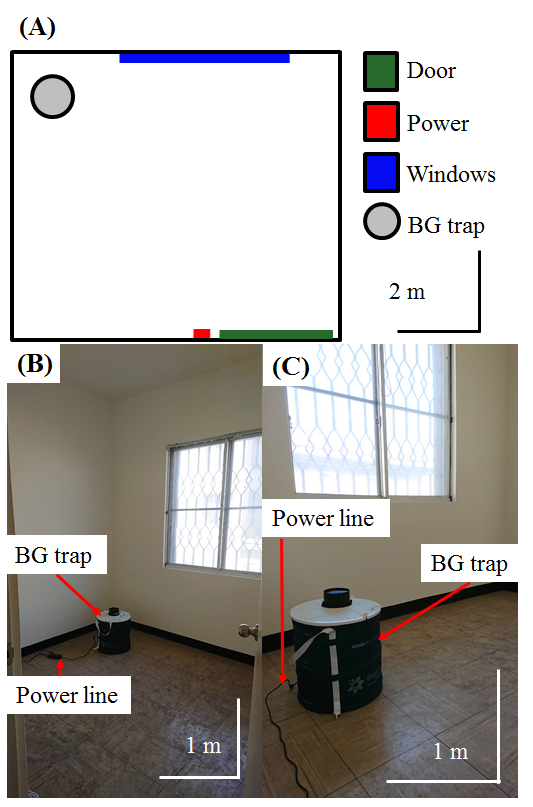


**Figure S8. Performance tests of smart mosquito traps integrated into BG traps in a small, simulated living room.** (**A**) The room layout and location where the combined BG trap and smart mosquito trap was placed for capture testing. (**B-C**) Actual photos of the room and the actual placement of the BG trap. No furniture or air conditioning was placed in the room to allow maximum space for the mosquitoes; the window remained closed. After the mosquito trap was activated, 50 mosquitoes were released into the room. The mosquitoes were allowed to fly freely in the room, and the trap captured the mosquitoes. After releasing the mosquitoes into the room, the sleeves of the insect cage were closed. The mosquitoes were allowed to fly freely in the limited space and were caught by the trap in each experiment.

**Supplementary Video (Movies)**

**Movie S1.** The establishment of the mosquito image database.

**Movie S2.** Dynamic mosquito image collection mechanism.

**Movie S3.** The dynamic mosquito studio inside perspective view.

**Movie S4.** Shoot and recognize the *Cx. quinquefasciatus* in the dynamic mosquito studio.

**Movie S5.** Shoot and recognize the *Ae. aegypti* in the dynamic mosquito studio.

**Movie S6.** The core device of the trap to identify and classify the mosquitoes.

**Movie S7.** The camera recorded the mosquitoes' flight status and mosquito trap status and classified the images as right video images.

**Movie S8.** The camera recorded the mosquitoes' flight status and mosquito trap status and classified the images as left video images.
